# Patient's Perspective on Using Mobile Technology as an Aid to Psychotherapy

**DOI:** 10.2196/10015

**Published:** 2018-10-24

**Authors:** Samantha Cristol

**Affiliations:** 1 William James College Newton, MA United States

**Keywords:** mobile phone app, technology, patient perspective

## Abstract

This piece draws from a patient’s perspective on his treatment using mobile health technology in conjunction with weekly group and individual psychotherapy. Research has demonstrated that using telepsychology as part of mental health treatment shows great promise to help advance the field of psychotherapy. Using mobile health technology such as mobile phone apps allows for collaboration with patients and their providers. This was written after several consultations with an individual diagnosed with borderline personality disorder who prefers to remain anonymous but was forthcoming with information regarding his use of mobile health technology in order to benefit the field of mental telepsychology.

## Introduction

This piece draws from a patient’s perspective on his treatment using mobile health technology in conjunction with weekly group and individual psychotherapy. Research has demonstrated that using telepsychology as part of mental health treatment shows great promise to help advance the field of psychotherapy. However, limited research has been done on mobile health apps. Despite the minimal research in this area, there are currently more than 10,000 mental health apps [1]. Using mobile health technology such as mobile phone apps allows for collaboration with patients and their providers. Furthermore, research has found no difference in the effectiveness between traditional face-to-face psychotherapy and therapeutic interventions that use the internet in some form [2].

## Brief Case

Alex Jones is a 30-year-old man who was diagnosed with both borderline personality disorder and major depressive disorder approximately 6 years ago in 2012. Prior to 2012, Alex graduated from college with a degree in art. After graduating from college, Alex worked for a few years as an art teacher at a local elementary school in Massachusetts; however, in the winter of 2012 he noticed his overall well-being started to decline. At this point, Alex began seeing a psychologist weekly for individual psychotherapy appointments and a psychiatrist monthly in order to sustain a beneficial medication regime.

In 2015, Alex had his first inpatient hospitalization following a suicide attempt. For the next 2 years, Alex had several inpatient hospitalizations and subsequently attended several day treatment programs. By 2017, Alex was “fed up” with his constant mood changes, suicidal ideation, and lack of relationships. During Alex’s intake appointment at a day treatment program, he made the following statement:

It’s annoying that I never feel the same way. I wake up and have no idea what I will feel that day. I know this is impacting my life, particularly, my relationships, but I don't know what to do about it.

Alex’s main goal at the time of this intake was to understand how to better track his mood symptoms with the ultimate goal of reviewing patterns in and consequences of his mood.

In the fall of 2017, Alex was attending at partial hospitalization program that used dialectical behavior therapy (DBT) [3], an evidenced-based therapy created for individuals diagnosed with borderline personality disorder. DBT targets patterns of behavior that are not helpful, such as self-harm, suicidal ideation, substance abuse, and poor relationships. As part of the DBT treatment, clients are asked to use a diary card in order to track their daily symptoms and urges. However, Alex did not like using the diary card because he felt it was inconvenient to carry around and would draw attention if he used it in a public place.

It’s embarrassing. I’m a 30-year-old man and yet I have to carry around a notebook like a child. People look at me weird when I randomly take out a notebook and I don't want to deal with that.

Alex and his therapist began to explore how mobile health technology might better suit his needs. As a result of these conversations, Alex began testing different mental health apps on his mobile phone. He felt that this was more subtle than taking out a notebook, primarily because other people did not know what he was using his mobile phone for. In order to determine which app Alex felt best fit his needs, he began by searching on “depression” in the App Store on his iPhone. The first app Alex tried was Happify, which provides individuals with tools and strategies that help with dysregulation. Alex did not find this app helpful because he was not looking for treatment strategies but rather a way to simply track his mood. Upon consulting with one of his friends, who also regularly sees a psychologist, Alex tried Moodtrack Social Diary Card. This app allows individuals to graph their moods. In addition, the app has a group chat feature that allows users to talk to one another about what they are experiencing. However, the app requires users to pay in order to keep their journal private. Given that Alex wanted to keep his identity on the app anonymous and was unwilling to pay for the app, the Moodtrack Social Diary Card was not a good fit.

Given that Alex did not like these first 2 apps, he searched again in the App Store on his iPhone and discovered the Daylio app [4]. This mobile health app is free and allows users to keep a private diary. On Daylio, users are asked to enter a quick, 2-step entry picking their mood and adding activities they have done throughout the day. The app also has a statistics and calendar section that allows users to better understand their patterns and habits. Finally, Daylio provides a section where users can write notes; in this section, Alex recorded what DBT skills he used that day. Alex chose to use this mobile health app because it was free, intuitive, and allowed for easy analysis of data. Alex and his therapist determined that Daylio best fit Alex’s current needs (ie, tracking mood, daily activities, and skills used each day). In the spring of 2017, Alex began regularly using Daylio and found that as he used it more consistently, it allowed for more collaboration with his providers.

Alex found using Daylio to be informative, which resulted in him becoming more engaged during therapy sessions. Alex eagerly shared the information from the Daylio app at each appointment with his therapist and together they used the data in order to inform treatment decisions. Because Alex used the app daily, it allowed him to discover his triggers and how he typically responded to those triggers. In addition, Alex was surprised by how frequently he was using DBT skills and how they actually helped his mood throughout the day. Based on this information, Alex felt he had more ownership over the DBT skills, which resulted in him becoming more engaged in therapy and more connected with other patients in the partial hospitalization program and helped Alex feel he would be ready for discharge from the program at an earlier date than scheduled.

## Brief Discussion

Alex’s case is important because it demonstrates how using mobile health apps can be beneficial in psychotherapy, both for clients and their providers. After the use of a mental health app, Alex, a client typically unengaged in therapy, became an active participant in his treatment. Having a free, easy-to-use, consistent tracking device allowed Alex to take ownership of his triggers, reactions, and symptoms. Furthermore, Alex was able to concretely see how frequently he was putting DBT skills to use in everyday life. While Alex is just one individual case and therefore cannot be generalized to all individuals with the same diagnoses, the main themes of Alex’s case are of significance to clients and providers alike.

Alex’s case shows how technology can aid in minimizing mental health stigma. Originally, Alex did not want to use a diary card (or other tracking device) because he was ashamed that individuals would see him using it and judge him for needing mental health treatment. According to Corrigan [5], mental illness stigma often leads to individuals opting out of or not fully participating in psychotherapy. Once Alex discovered that using Daylio was subtle, as people just “thought I was on my phone doing whatever,” he felt more comfortable implementing it as part of his everyday routine.

Furthermore, Alex’s case underscores how the use of mental health apps allows for more collaboration between clients and their providers. Prior to using the Daylio app, Alex and his provider were frequently not on the same page in terms of treatment goals. However, once Alex began consistently using the Daylio app to track his mood, daily activities, and skill use, he and his therapist were able to collaborate to create common goals based on concrete data the app generated. Alex stated he felt more “heard” and “validated” after using Daylio because he and his therapist could visually examine how his week went and use treatment techniques that would encourage a more positive response in the future.

Finally, this case demonstrates that technology can be used in conjunction with psychotherapy rather than the either/or approach frequently seen. For Alex, technology provided him the data that was necessary to both inform treatment and increase his self-awareness. The decision to use an app only occurred after a collaborative conversation between Alex and his therapist in which multiple options were reviewed in order to satisfy Alex’s current need. In this case, technology was successfully used to augment a pre-existing treatment plan. As mental health apps continue to be developed, it is important to use the therapeutic alliance to help patients match app functions with their needs and treatment plans.

## Abbreviations

DBT: dialectical behavior therapy
